# Comparing Guillain-Barré syndrome outcomes between rural and urban hospitals in the United States: A retrospective cohort study

**DOI:** 10.1371/journal.pone.0333403

**Published:** 2025-09-25

**Authors:** Anudeep Surendranath, Rahul Damani, Anushareddy Muddasani, Anupam Kumar Gupta

**Affiliations:** 1 CHI St. Vincent – Hot Springs, Hot Springs, Arkansas, United States of America; 2 Baylor College of Medicine Medical Center, McNair Campus, Houston, Texas, United States of America; 3 University of Arkansas for Medical Sciences, Little Rock Arkansas, United States of America; 4 SSM Heath, Mt Vernon, Illinois, United States of America; Creighton University, UNITED STATES OF AMERICA

## Abstract

**Background and purpose:**

Rural-urban disparities in neurological care have been well documented, but limited data exist regarding Guillain-Barré Syndrome (GBS). This study examines differences in patient demographics, hospital characteristics, and outcomes among GBS admissions to rural versus urban hospitals in the United States.

**Methods:**

Using the 2021 National Inpatient Sample, we conducted a retrospective cohort study of adult hospitalizations with a principal diagnosis of GBS. Hospitals were classified as rural or urban based on U.S. census designations. Multivariate logistic and linear regression models were used to assess associations between hospital location and outcomes, adjusting for demographic, clinical, and hospital-level factors.

**Results:**

An estimated 10,035 weighted Guillain-Barré Syndrome hospitalizations were identified, of which 95.8% occurred in urban hospitals. Rural hospitalizations involved older individuals (mean age 56.8 years; 95% CI: 52.5–61.0) compared to urban hospitalizations (51.3 years; 95% CI: 50.3–52.2). Adjusted analyses showed no significant differences in in-hospital mortality (adjusted OR 2.00; 95% CI: 0.11–35.12) or length of stay (mean difference −1.85 days; 95% CI: −6.62 to 2.91). However, total hospital charges were significantly higher in urban hospitals, with an average difference of $39,474 (95% CI: $4,296–$74,651). Discharge disposition was comparable, with 40% of rural hospitalizations and 48.1% of urban hospitalizations discharged home, and 38.8% versus 43.3% discharged to skilled nursing facilities (all p > 0.05).

**Conclusions:**

In this national analysis of over 10,000 Guillain-Barré Syndrome hospitalizations, rural and urban hospitals achieved comparable outcomes in terms of in-hospital mortality, length of stay, complications, and discharge disposition. Rural hospitalizations tended to involve older individuals from lower-income areas, whereas urban hospitals managed more cases with severe comorbidities and generated substantially higher costs. These findings suggest that rural hospitals are capable of delivering effective acute care for GBS, and highlight the need for future research on long-term functional outcomes across geographic settings.

## 1. Introduction

Guillain-Barré Syndrome (GBS) is a classically postinfectious condition causing progressive weakness that typically starts in the distal extremities and may involve cranial and respiratory muscles. It is the most common cause of acute flaccid paralysis [1]. It presents with progressive, symmetric limb weakness, which may be accompanied by sensory symptoms. Examination typically shows hyporeflexia or areflexia, and cerebrospinal fluid reveals elevated protein with normal or mildly increased white cell count [2]. The symptoms usually reach a maximum severity within 4 weeks of symptom onset [3].

Several variants of GBS have been identified based on electrophysiological characteristics, including acute inflammatory demyelinating polyneuropathy (AIDP), acute motor axonal neuropathy (AMAN), and acute motor and sensory axonal neuropathy (AMSAN) [4]. The International Guillain-Barré Syndrome Outcome Study highlighted significant regional differences in the clinical presentation and outcomes of Guillain-Barré Syndrome. For example, sensorimotor GBS was more common in Europe/Americas, pure motor GBS predominated in Bangladesh, and Miller Fisher syndrome was more frequent in Asia. These variations underline the influence of geography on disease phenotype and prognosis [5].

Plasma exchange (PE) and intravenous immunoglobulin (IVIg) are established treatments for Guillain-Barré Syndrome, with studies showing similar efficacy between these modalities in improving disability [6]. Despite the availability of intravenous immunoglobulin (IVIg) and plasma exchange (PLEX) as standard treatments, Guillain-Barré Syndrome (GBS) remains associated with significant mortality and long-term morbidity. In the acute phase, up to 22% of patients require mechanical ventilation, which is a key driver of ICU admission and a predictor of subsequent disability [7]. In-hospital mortality specifically ranges from 2.8% to 4.4%, with higher rates observed in patients admitted to intensive care units (ICUs), where mortality can exceed 7%, compared to less than 2% in patients managed outside the ICU [8]. Autonomic dysfunction, including cardiac arrhythmias and blood pressure instability, is also a common and serious complication during this phase [7]. Mortality rates are reported at 2.8% within 6 months and increase to 3.9% at 12 months of observation [8]. Most deaths occur during the recovery phase following initial neurological improvement and are frequently caused by pneumonia, sepsis, or cardiovascular events, including myocardial infarction and severe autonomic dysfunction [8]. Advanced age, severe weakness at onset, and delayed treatment are critical predictors of mortality [9]. Although many patients recover, approximately 14% experience severe disability at one year, with persistent deficits or incomplete recovery affecting a significant proportion [10].

In contrast to other neurological conditions such as stroke, where outcome disparities between rural and urban hospitals are well documented, [11] the literature on such differences in Guillain-Barré Syndrome (GBS) remains sparse. To address this knowledge gap, we aimed to evaluate these differences using the National Inpatient Sample (NIS) database, leveraging its extensive coverage and robust methodology to provide insights into the impact of hospital location on GBS outcomes.

## 2. Methods

### 2.1 Study design and database description

This retrospective cohort study utilized the 2021 National Inpatient Sample (NIS), a publicly available and fully de-identified database developed by the Healthcare Cost and Utilization Project (HCUP) under the Agency for Healthcare Research and Quality (AHRQ). The NIS compiles data from 48 states, covering 97% of the U.S. population and 96% of discharges from community hospitals, excluding long-term acute care and rehabilitation facilities. It represents a 20% stratified sample of discharges designed to reflect hospital characteristics such as ownership, teaching status, location, and bed size. Its self-weighting methodology ensures reliable national estimates while protecting patient privacy by omitting all personal identifiers, including state and hospital information. Authors accessed the data for research purposes on April 14, 2025, and did not have access to any identifiable patient information during or after data collection. As the NIS is both publicly available and de-identified, Institutional Review Board (IRB) approval and informed consent were not required. The large sample size and national scope of the NIS provide robust statistical power to examine variations in patient characteristics, hospital types, and clinical outcomes.

### 2.2 Study population

Hospitalizations were identified through ICD-10 codes (provided in the supplementary table). Admissions were categorized based on the hospital type—rural or urban. Urban hospitals were defined as those located in counties classified as Metropolitan Statistical Areas (MSAs), which consist of a core urban area with a population of 50,000 or more and demonstrate significant social and economic integration with surrounding counties. Rural hospitals were in counties classified as Micropolitan Statistical Areas (urban clusters with populations between 10,000 and 49,999) or in areas outside any Core-Based Statistical Area (CBSA). This binary classification follows the CBSA-based definitions used by the Healthcare Cost and Utilization Project (HCUP) in the NIS and aligns with the Office of Management and Budget (OMB) criteria. While this approach ensures consistency with the way hospital location is coded in the NIS, it is less granular than the six-level urban-rural classification developed by the National Center for Health Statistics (NCHS). The NCHS Urban-Rural Classification Scheme for Counties, often referred to as the PL_NCHS scheme, categorizes counties into six groups based on population size and proximity to metropolitan areas, providing more detailed insights into rurality. This trade-off was accepted to maintain compatibility with the structure of the NIS dataset and to allow for interpretable, nationally representative comparisons.

### 2.3 Outcomes

The primary outcome of interest was in-hospital mortality. Secondary outcomes included total hospital charges and total length of stay.

### 2.4 Statistical analysis

The data analysis was performed using Stata software, version 18. Continuous variables were analyzed using t-tests to determine p-values, whereas Fisher’s exact test was utilized for binary and categorical data. To evaluate the relationship between hospital location and binary outcomes, multivariate logistic regression was employed, while multivariate linear regression was used for continuous outcomes. Both regression models were adjusted for the following covariates: age, sex, race/ethnicity, insurance status, median household income, hospital region, teaching status, Charlson Comorbidity Index (CCI), and intubation/mechanical ventilation. Stata excludes observations with missing data by default during regression and summary analyses. As such, a complete case analysis was performed.

## 3. Results

### 3.1 Patient demographics and hospital characteristics

A total of 10,035 hospitalizations with a principal diagnosis of Guillain-Barré Syndrome (GBS), of whom 95.8% (n = 9,610) were admitted to urban hospitals. This disparity in admission volume likely reflects national population patterns, as over 80% of the U.S. population resides in urban areas as defined by the U.S. Census Bureau and used in NIS classification [12]. Because NIS records only the hospital of discharge, patients transferred from rural to urban facilities are attributed to urban hospitals. Table 1 summarizes the differences in demographic and hospital characteristics between rural and urban hospitalizations.

**Table 1:** Patient demographics and hospital characteristics.

	Rural	Urban	P value
Female (%)	205 (48.24)	4575 (47.63)	0.91
Mean age (95% conf. interval)	56.79 (52.52-61.05)	51.29 (50.33-52.25)	0.01
Race/ethnicity (%)			0.09
White	325 (84.42)	6310 (68.22)	
Black	20 (5.19)	1045 (11.3)	
Hispanic	30 (7.79)	1285 (13.89)	
Asian or Pacific Islander	0 (0)	235 (2.54)	
Native American	5 (1.3)	100 (1.08)	
Other	5 (1.3)	275 (2.97)	
Median annual income in patient’s zip code, US$, no. (%)			0.00
$1 - $51,999	220 (54.32)	2430 (25.67)	
$52,000 - $65,999	125 (30.86)	2270 (23.98)	
$66,000 - $87,999	55 (13.58)	2400 (25.36)	
$88,000 or more	5 (1.23)	2365 (24.99)	
Insurance type, no. (%)			0.21
Medicare	175(42.68)	2975 (32.04)	
Medicaid	65 (15.85)	1815 (19.55)	
Private insurance, including Health Maintenance Organizations (HMO)	145 (35.37)	4075 (43.89)	
Self-pay	25 (6.1)	420 (4.52)	
Hospital region, no. (%)			0.00
Northeast	35 (8.24)	1495 (15.56)	
Midwest	185 (43.53)	2085 (21.7)	
South	155 (36.47)	3805 (39.59)	
West	50 (11.76)	2225 (23.15)	
Teaching hospital	0 (0)	8120 (84.5)	0.00
Charlson Comorbidity Index score, no. (%)			0.01
0	140 (32.94)	4380 (45.58)	
1	115 (27.06)	1810 (18.83)	
2	110 (25.88)	1585 (16.49)	
≥3	60 (14.12)	1835 (19.09)	

There was no significant difference in gender distribution between the two groups, with females comprising 48.24% of rural admissions and 47.63% of urban admissions (p = 0.91). However, the mean age was significantly higher among rural hospitalizations (56.79 years; 95% CI: 52.52–61.05) compared to urban hospitalizations (51.29 years; 95% CI: 50.33–52.25; p = 0.01).

Socioeconomic status, as indicated by the median income in patients’ zip codes, varied significantly (p < 0.01). Over half of rural hospitalizations (54.32%) occurred among individuals residing in areas with a median income below $51,999, compared to only 25.67% of urban hospitalizations. In contrast, 24.99% of urban hospitalizations were from higher-income zip codes ($88,000 or more), compared to just 1.23% in the rural cohort.

Racial and ethnic distributions trended toward significance (p = 0.09), with a greater proportion of White hospitalizations in rural hospitals (84.42%) compared to urban hospitals (68.22%). Meanwhile, urban hospitals had a higher representation of Hispanic hospitalizations (13.89% vs. 7.79%) and Black hospitalizations (11.3% vs. 5.19%).

Insurance coverage did not differ significantly between groups (p = 0.21). Medicare was the most common payer in both settings (42.68% rural vs. 32.04% urban), followed by private insurance and Medicaid.

Geographic distribution varied significantly (p < 0.01). The majority of rural GBS admissions were located in the Midwest (43.53%), while urban admissions were more common in the South (39.59%). All rural hospitalizations occurred at non-teaching facilities, whereas 84.5% of urban hospitalizations took place in teaching hospitals (p < 0.01).

Comorbidity burden, as measured by the Charlson Comorbidity Index (CCI), also differed significantly (p = 0.01). Rural hospitals had a higher proportion of hospitalizations with moderate comorbidities (CCI scores of 1 or 2), while urban hospitals had a greater share of hospitalizations with no comorbidities (45.58% vs. 32.94%) or severe comorbidities (CCI ≥ 3: 19.09% vs. 14.12%).

### 3.2 Primary outcome

Among the 10,035 hospitalizations with a principal diagnosis of Guillain-Barré Syndrome (GBS), the overall in-hospital mortality rate was 1.44%. Unadjusted analysis showed no significant difference in mortality between urban and rural hospitals (odds ratio [OR] 2.16; 95% confidence interval [CI]: 0.21–21.88; p = 0.51). After adjusting for age, sex, race/ethnicity, insurance status, median household income, hospital region, teaching status, Charlson Comorbidity Index (CCI), and intubation/mechanical ventilation, the adjusted odds ratio for mortality associated with urban hospital admission was 2.00 (95% CI: 0.11–35.12; p = 0.63). Similar findings were obtained when adjusting for only those variables with a p-value < 0.2 in the univariate analysis, further validating the lack of significant difference in hospital mortality between the two settings. The primary outcome results are summarized in Table 2.

**Table 2:** Primary outcome.

Outcome	Hospital location	Odds Ratio (OR)	95% Confidence Interval (CI)	p-value
Mortality (Unadjusted)	Urban	2.16	0.21–21.88	0.51
Mortality (Adjusted)	Urban	2.00	0.11–35.12	0.63

### 3.3 Secondary outcomes

The adjusted analysis of secondary outcomes revealed no statistically significant differences in length of stay (LOS) between rural and urban hospitals. On average, patients admitted to rural hospitals had a 1.85-day shorter LOS compared to urban hospitals (95% confidence interval [CI]: −6.62 to 2.91; p = 0.44).

Adjusted total hospital charges were significantly higher in urban hospitals, with an average difference of $39,474 (95% CI: $4,296 to $74,651; p = 0.03). These models accounted for demographic, clinical, hospital-level factors, and mechanical ventilation to better reflect disease severity.

These findings suggest that while LOS remained similar across settings, hospitalizations in urban settings were associated with significantly higher costs. Detailed results are presented in Table 3.

**Table 3:** Secondary outcomes.

Adjusted Secondary Outcomes	Value	95% Confidence Interval (CI)	p-value
Mean Length of Stay (Urban)	1.85 days shorter	−6.62–2.91 days	0.44
Total Hospital Charges (Urban)	$39,474 higher	$4296 - $74,651	0.03

### 3.4 Patient complications

To further evaluate disease severity and potential contributors to in-hospital outcomes, we analyzed the frequency of key complications associated with Guillain-Barré Syndrome (GBS), including respiratory failure requiring mechanical ventilation, sepsis, motor deficits, and autonomic dysfunction. These results are summarized in Table 4.

**Table 4:** Patient complications.

Patient Complications	Rural	Urban	p-value
Intubation and mechanical ventilation (%)	35 (8.24)	840 (8.74)	0.86
Sepsis (%)	10 (2.35)	320 (3.33)	0.62
Paraplegia/paraparesis(%)	5 (1.18)	345 (3.59)	0.24
Quadriplegia/quadriparesis (%)	20 (4.71)	685 (7.13)_	0.36
Autonomic dysfunction (%)	60 (14.12)	1055 (10.98)	0.43

Rates of intubation and mechanical ventilation were similar between rural (8.24%) and urban (8.74%) hospitals (p = 0.86), suggesting comparable levels of respiratory compromise requiring critical care support. The incidence of sepsis was also not significantly different (2.35% rural vs. 3.33% urban; p = 0.62). Motor complications such as paraplegia/paraparesis and quadriplegia/quadriparesis were slightly more frequent among urban patients (3.59% and 7.13%, respectively) than rural patients (1.18% and 4.71%, respectively), although these differences did not reach statistical significance (p = 0.24 and p = 0.36, respectively). Interestingly, autonomic dysfunction appeared more common in rural admissions (14.12%) compared to urban settings (10.98%), though again without statistical significance (p = 0.43).

Overall, there were no significant rural-urban differences across any of the examined complications, reinforcing the primary finding of comparable in-hospital outcomes between the two hospital types.

### 3.5 Discharge disposition

As shown in Table 5, there were no statistically significant differences in discharge outcomes between rural and urban hospitals. Among hospitalizations with Guillain-Barré Syndrome, 40% of rural hospitalizations were discharged home compared to 48.07% in urban hospitals (p = 0.15). Similarly, discharge to skilled nursing facilities occurred in 38.82% of rural hospitalizations and 43.29% of urban hospitalizations (p = 0.42). These findings suggest broadly comparable discharge patterns across hospital settings, despite demographic and hospital-level differences. The absence of statistically significant variation indicates that both rural and urban hospitals achieved similar post-acute care dispositions for GBS patients.

**Table 5:** Discharge disposition.

Discharge disposition	Rural	Urban	p-value
Home (%)	170 (40)	4620 (48.07)	0.15
Skilled Nursing Facility (%)	165 (38.82)	4160 (43.29)	0.42

## 4. Discussion

In this national analysis of 10,035 Guillain-Barré Syndrome (GBS) hospitalizations, we found no statistically significant differences between rural and urban hospitals in in-hospital mortality, hospital length of stay, or complication rates. However, total hospital charges were significantly higher in urban hospitals, even after adjusting for demographic, clinical, and hospital-level factors, including severity of illness as indicated by mechanical ventilation. Rural hospitalizations involved older individuals and were more likely to originate from lower-income areas, while urban hospitals had a higher proportion of hospitalizations with severe comorbidities. Complication rates, including intubation, sepsis, motor deficits such as paraplegia or quadriplegia, and autonomic dysfunction, remained similar across both settings. These findings suggest that, despite differences in patient populations and hospital characteristics, rural and urban hospitals achieved comparable clinical outcomes for GBS hospitalizations, although care in urban settings was associated with greater financial cost.

Rural-urban disparities in healthcare outcomes have been well-documented in acute stroke care. Hammond et al. found that rural stroke patients were less likely to receive advanced treatments, such as thrombolysis and endovascular therapy, resulting in higher in-hospital mortality compared to urban patients [11]. These differences persisted over a five-year period, reflecting structural inequities in access to evidence-based care. Similarly, Wilcock et al. reported that although access to certified stroke centers improved in rural areas over time, disparities in the use of advanced therapies like alteplase remained, highlighting ongoing systemic barriers in rural healthcare delivery [13]. However, despite this body of literature in stroke, data on rural-urban differences in Guillain-Barré Syndrome (GBS) outcomes have been lacking in developed countries. In contrast to the findings in stroke, our study found no statistically significant difference in in-hospital mortality between rural and urban hospitals for GBS admissions, suggesting that geographic disparities may have a more limited impact on acute outcomes in this condition.

There were no statistically significant differences in mortality or length of stay between rural and urban hospitals in our study. However, hospital charges were significantly higher in urban settings. These finding challenges assumptions that resource limitations in rural settings necessarily translate to worse outcomes or higher costs. While prior studies have emphasized gaps in access to specialty care and advanced interventions in rural hospitals [14], our results suggest that, at least for acute GBS management, such disparities may not significantly affect short-term hospital-based outcomes. Nevertheless, existing literature continues to document differences in therapy utilization between rural and urban patients for other neurologic conditions, raising questions about whether rural patients with GBS may still face barriers to timely diagnosis or advanced care [15].

The lack of mortality difference in our study is notable given the expectation that limited critical care capacity in rural settings might negatively impact outcomes. Our findings may reflect either adequate triaging and transfer systems for high-risk patients or a true parity in acute management of Guillain-Barré Syndrome (GBS). Our findings are further supported by international studies. In a multicenter retrospective analysis from southern China, Zhou et al. reported no significant rural-urban differences in demographics, clinical features, disease progression, or cerebrospinal fluid findings among hospitalized GBS patients. Mechanical ventilation rates were slightly higher in urban patients (10.94% vs. 8.65%), and seasonal variation was noted in both settings, but these differences were not statistically significant [16]. Similarly, Govoni et al. found a higher incidence of GBS in urban centers compared to rural areas in northern Italy, although early clinical outcomes were not substantially different [17] .These findings from diverse healthcare systems reinforce our observation that acute-phase GBS care may yield comparable outcomes across geographic settings, even in the context of varying incidence or resource distribution. In contrast, studies have shown higher mortality in low-resource countries and in rural populations within other global settings, often attributed to delays in presentation, lack of ICU availability, and limited access to immunotherapy [18].

Notably, previous studies have established that neurologist density is unevenly distributed across the U.S., with urban areas having higher specialist availability and rates of neurologist involvement in care [19]. Although urban hospitals may be more likely to provide advanced therapies, our findings suggest that the increased access does not necessarily translate into improved survival. This supports prior observations that higher healthcare spending does not always equate to better patient outcomes [20].

The observed in-hospital mortality rate of 1.44% in our study is lower than the 2.8–4.4% reported in prior studies of Guillain-Barré Syndrome [8]. Several factors may explain this discrepancy. First, improvements in early diagnosis, critical care, and access to immunomodulatory therapies may have contributed to improved survival in recent years. Second, our study utilizes a nationally representative dataset that includes hospitals of all sizes and capabilities, whereas earlier mortality estimates such as those reported by van den Berg et al. were based on pooled data from three Dutch studies [21–23]. All three studies were conducted at tertiary care academic centers, which typically manage more complex and critically ill cases. This likely contributed to higher observed mortality due to referral and severity bias.

From a public health standpoint, the economic burden of GBS is substantial and disproportionately driven by indirect costs, including productivity loss and long-term disability [24]. Ensuring early recognition and timely initiation of immunomodulatory therapy remains essential, particularly in resource-limited settings. Delays in diagnosis, especially among patients with overlapping neurological conditions or atypical presentations, have been shown to negatively affect timely management of GBS. A study by Pathikonda et al. found that motor-sensory or autonomic symptoms, and comorbid neurologic diseases, were associated with longer diagnostic delays. These factors may disproportionately affect rural populations, where access to neurology consultation is limited, potentially influencing treatment decisions even when mortality remains unaffected. [25].

Our study contributes novel national-level insights into rural-urban disparities in GBS care, a topic previously underexplored. Unlike the extensive literature on stroke and other neurologic diseases that demonstrate poorer rural outcomes, our findings highlight the need to nuance assumptions about rural disadvantage. Future studies should investigate long-term functional outcomes and access to rehabilitation services, which are likely influenced by geography and social determinants of health. In addition, conducting similar studies using multiple years of NIS data may help improve generalizability and allow for assessment of trends over time.

This study has several limitations. It is based on the National Inpatient Sample (NIS), which uses administrative coding at the discharge level and relies on accurate documentation. Although the dataset is robust and nationally representative, misclassification of diagnoses cannot be ruled out. As a sample rather than a census, some patient- and hospital-level details may be underrepresented, which could limit generalizability. The NIS also does not contain data on medications, imaging studies, or laboratory results, restricting the ability to analyze associations with specific treatments. The high proportion of GBS admissions in urban hospitals (95.8%) likely reflects the population distribution, since over 80 percent of the U.S. population resides in urban areas as defined by the U.S. Census Bureau and used by the NIS classification system [12]. Because NIS records only the hospital of discharge, patients transferred from rural to urban facilities are attributed to urban hospitals. Lastly, as a retrospective observational study, the analysis cannot establish causal relationships.

## 5. Conclusion

In this national analysis of over 10,000 Guillain-Barré Syndrome (GBS) hospitalizations, we found that rural and urban hospitals achieved broadly comparable outcomes with respect to in-hospital mortality, length of stay, complications, and discharge disposition. Rural hospitalizations more often involved older individuals from lower-income areas, whereas urban hospitals managed a greater proportion of hospitalizations with severe comorbidities and were associated with substantially higher hospital charges. These findings challenge the assumption that rural hospitals deliver inferior acute neurologic care and suggest that, for GBS, geography may play a limited role in short-term inpatient outcomes. Nonetheless, the marked difference in healthcare costs across settings underscores the importance of evaluating resource utilization. Future work should focus on long-term outcomes and multi-year analyses to better understand geographic disparities in GBS care and inform equitable allocation of healthcare resources.

## Supporting information

S1 Table ICD-10 codes. This supplementary file contains the complete list of ICD-10 codes used to identify hospitalizations from the National Inpatient Sample. (DOCX)
