# Supplementary material for: Comparing Guillain-Barré syndrome outcomes between rural and urban hospitals in the United States: A retrospective cohort study
Source: PLoS One. 2025 Sep 25;20(9):e0333403. doi: 10.1371/journal.pone.0333403 (PMC12463200; doi:10.1371/journal.pone.0333403)
Supplement: S1 Table — (DOCX) [file pone.0333403.s001.docx]

| **Diagnosis** | **ICD-10 codes** |
| --- | --- |
| GBS | G610 |
| Sepsis | A419 A415 A4151 A4152 A418 A4181 A4189 R6520 R6521 |
| Paraplegia/paraparesis | G8220 G8221 G8222 G8223 |
| Quadriplegia/quadriparesis | G8250 G8251 G8252 G8253 |
| Autonomic Dysfunction: | G900 G9009 R338 R339 I499 I951 |
| Intubation/mechanical ventilation | 0BH17EZ, 0BH18EZ, 0B717DZ, 0B718DZ, 0BH07DZ, 0BH07YZ, 0BH172Z, 0BH17YZ, 0BH182Z, 0BH18YZ, 0BHK7YZ, 0BHK8YZ, 0BHL7YZ, 0BHL8YZ, 0WHQ7YZ, 5A1935Z, 5A1945Z, 5A1955Z |
